# DNA Vaccine Encoding the Chimeric Form of *Schistosoma mansoni* Sm-TSP2 and Sm29 Confers Partial Protection against Challenge Infection

**DOI:** 10.1371/journal.pone.0125075

**Published:** 2015-05-05

**Authors:** Natan Raimundo Gonçalves de Assis, Suellen Batistoni de Morais, Bárbara Castro Pimentel Figueiredo, Natasha Delaqua Ricci, Leonardo Augusto de Almeida, Carina da Silva Pinheiro, Vicente de Paulo Martins, Sergio Costa Oliveira

**Affiliations:** 1 Departamento de Bioquímica e Imunologia do Instituto de Ciências Biológicas, Universidade Federal de Minas Gerais, 31270–901, Belo Horizonte, MG, Brazil; 2 Instituto Nacional de Ciência e Tecnologia em Doenças Tropicais (INCT-DT), CNPq MCT, 31270–901, Belo Horizonte, MG, Brazil; 3 Departamento de Biointeração do Instituto de Ciências da Saúde, Universidade Federal da Bahia, 40110–100, Salvador, BA, Brazil; 4 Faculdade de Ceilândia, Universidade de Brasília, 70910–900, Brasília, DF, Brazil; Federal University of São Paulo, BRAZIL

## Abstract

Schistosomiasis is an important parasitic disease worldwide that affects more than 207 million people in 76 countries and causes approximately 250,000 deaths per year. The best long-term strategy to control schistosomiasis is through immunization combined with drug treatment. Due to the ability of DNA vaccines to generate humoral and cellular immune responses, such vaccines are considered a promising approach against schistosomiasis. Sm29 and tetraspanin-2 (Sm-TSP2) are two proteins that are located in the *S*. *mansoni* tegument of adult worms and schistosomula and induce high levels of protection through recombinant protein immunization. In this study, we transfected BHK-21 cells with plasmids encoding Sm29, Sm-TSP2 or a chimera containing both genes. Using RT-PCR analysis and western blot, we confirmed that the DNA vaccine constructs were transcribed and translated, respectively, in BHK-21 cells. After immunization of mice, we evaluated the reduction in worm burden. We observed worm burden reductions of 17-22%, 22%, 31-32% and 24-32% in animals immunized with the pUMVC3/Sm29, pUMVC3/SmTSP-2, pUMVC3/Chimera and pUMVC3/Sm29 + pUMVC3/SmTSP-2 plasmids, respectively. We evaluated the humoral response elicited by DNA vaccines, and animals immunized with pUMVC3/Sm29 and pUMVC3/Sm29 + pUMVC3/SmTSP-2 showed higher titers of anti-Sm29 antibodies. The cytokine profile produced by the spleen cells of immunized mice was then evaluated. We observed higher production of Th1 cytokines, such as TNF-α and IFN-γ, in vaccinated mice and no significant production of IL-4 and IL-5. The DNA vaccines tested in this study showed the ability to generate a protective immune response against schistosomiasis, probably through the production of Th1 cytokines. However, future strategies aiming to optimize the protective response induced by a chimeric DNA construct need to be developed.

## Introduction

Schistosomiasis is an important parasitic disease that affects approximately 207 million people in 76 countries worldwide, of which 120 million are symptomatic and 20 million develop the severe form of the disease [1]. Presently, the main strategy against schistosomiasis is chemotherapeutic treatment, but in spite of decades of treatment, high rates of reinfection and the emergence of strains resistant to chemotherapy make this approach alone inefficient [1–3]. Many investigators believe that a prophylactic vaccination would be an ideal approach either alone or together with chemotherapy, which can be effective in the reduction of morbidity and transmission of schistosomiasis [4,5]. A vaccine that induces even a partial reduction in worm burden could considerably reduce pathology and limit parasite transmission [6,7].

DNA vaccines are an approach that instead of using the antigen itself uses the DNA sequence encoding the antigen [8]. Further, DNA vaccines have the potential to induce innate and adaptive immune systems, are easy to manufacture and distribute and are stable at room temperature and apparently safe for human use, which are considered by many to be ideal characteristics of an effective vaccine against schistosomiasis [9–14]. Some DNA vaccines have already been tested against *S*. *mansoni*, and several of them have achieved significant levels of protection, such as Sm-p80, Sm23 and Cu/Zn superoxide dismutase, which showed 59%, 25–44% and 44–60% worm burden reduction, respectively [15–17].

In the scenario of developing a vaccine against *S*. *mansoni*, the proteins Sm29 and tetraspanin-2 (SmTSP-2) appear to be potential candidates [18–21]. Both Sm29 and TSP-2 are present in the tegument of adult worms and schistosomula, and when evaluated as a recombinant protein, they reached 51% and 57% protection, respectively [18,19]. Despite efforts to develop a vaccine against *S*. *mansoni*, an efficient commercial product has not yet been developed. This suggests a need for strategies to improve their efficacy, and one of these strategies is the use of two or more antigens, alone or in a chimeric form [22,23]. A work carried out by Jia and coworkers (2014) showed that Sm29 could be a partner of TSP-2 for generation of protein complexes called tetraspanin-enriched microdomains (TEMs), which seem to be essential to worm survival and are of particular importance for the development of a multivalent vaccine against schistosomiasis [24].

Herein using a mouse model of schistosomiasis, we evaluated the potential of DNA vaccines, which encoded the sequence for the proteins Sm29 and tetraspanin-2 alone, in a chimeric form or together.

## Material and Methods

### DNA vaccine constructs

To prepare the DNA vaccines, the sequence of recombinant Sm29 described earlier [25], the full length of extracellular loop 2 of SmTSP-2 [18] and a chimeric sequence, containing the same sequence of the recombinant Sm29 (Val27-Lys169) at the N-terminal portion, followed by the same sequence of the entire extracellular loop-2 of Sm-TSP-2 (Glu107-His184) at the C-terminal portion, were synthesized and inserted between the Eco*RI* and Xba*I* sites of the pUMVC3 plasmid (Aldevron, Fargo, ND, USA). These constructs were created and optimized by Epoch Life Sciences (Missouri City, Texas, USA), and the plasmids were designated pUMVC3/Sm29, pUMVC3/SmTSP-2 and pUMVC3/Chimera. A Kozak sequence (5’ GCCGCCACCATGG 3’) was inserted before each schistosome gene sequence, and the underlined codon represents the start codon [26,27]. Plasmid DNA purification was performed using an endotoxin free Giga prep kit according to the manufacturer’s instructions (Qiagen, Valencia, CA).

### Recombinant protein production

Recombinant Sm29, Chimeras A and B were produced and purified as described previously [25,28]. Briefly, the cDNAs sequences of Sm29 (Val27-Lys 169), chimera A (composed by the sequence of the extracellular loop-2 of Sm-TSP-2 at the N-terminal position, and the N-terminal sequence of rSm29 (Val27-Leu87) at the C-terminal position) and chimera B (composed by the extracellular loop-2 of Sm-TSP-2 at the N-terminal position, and the C-terminal sequence of rSm29 (Cys88-Lys169) at the C-terminal position), were fused with a C-terminal 6x histidine and produced in *E*. *coli* using the expression vectors pET21a for Sm29 or pET41a for recombinant chimera (Novagen, NJ, USA). Recombinant Sm29 and chimeras were purified in an affinity column and dialyzed against PBS pH 7.0.

Regarding the recombinant TSP-2, the sequence of extracellular loop of TSP-2 (Glu107-His184) was synthesized in pD444-CH plasmid by the company DNA 2.0. One liter of *E*.*Coli* BL21 (DE3 codon plus) culture containing the recombinant plasmid was grown at 30°C to an optical density of approximately 0.5–0.8 at 600nm and gene expression of rTSP-2 was induced using 1 mM IPTG. The rest of the protocol for rTSP-2 production is similar to the one used for rSm29 production as previously described [25]. The recombinant Sm29, TSP-2 and chimera (chimera A and B), were used as antigens for in vitro immunological assays.

### Evaluation of DNA vaccine mRNA transcripts in BHK-21 cells

The level of mRNA transcripts of Sm29, SmTSP-2 or chimera DNA vaccines was determined by real time RT-PCR in Baby Hamster Kidney-21 (BHK-21; ATCC catalog# CCL-10) [29] transfected cells. Briefly, BHK-21 cells were grown in RPMI medium in 24-well plates and transfected using 0.5 μg of pUMVC3/Sm29, pUMVC3/SmTSP-2, pUMVC3/chimera or the control (pUMVC3) using the transfecting agent Lipofectamine 2000 according to the manufacturer’s instructions (Invitrogen, Carlsbad, CA). After transfection, the cells were kept in RPMI medium for 16 hours at 37°C in 5% CO_2_. Then, total RNA was extracted using the Illustra RNAspin Mini kit (GE Healthcare, Buckinghamshire, UK) according to the manufacturer’s instructions. Reverse transcription of 1 μg of the total RNA was performed using illustra Ready-To-Go RT-PCR Beads (GE Healthcare, Buckinghamshire, UK). Real-Time RT-PCR was conducted using a final volume of 10 μL containing the following: SYBRH Green PCR Master Mix (Applied Biosystems, Foster City, CA), oligo-dT, cDNA as the PCR template and 20 μM primers. The PCR reaction was performed using an ABI 7900 Real-Time PCR System (Applied Biosystems, Foster City, CA) with the following cycling parameters: 60°C for 10 min, 95°C for 10 min, 40 cycles at 95°C for 15 sec and 60°C for 1 min, and a dissociation stage of 95°C for 15 sec, 60°C for 1 min, 95°C for 15 sec, and 60°C for 15 sec. Primers were designed using PRIMER3 and used to amplify a specific 100–120-bp fragment corresponding to specific optimized gene targets, as follows: β-Actin F: 5’-AGGTGTGCACCTTTTATTGGTCTCAA-3’; β-Actin R: 5’-TGTATGAAGGTTTGGTCTCCCT-3’; Sm29 F: 5’-CGGAATCCCCATAAACTTCC-3’; Sm29 R: 5’-CGGACAGCACTTTCTGGTTT-3’; SmTSP-2 F: 5’-ACATCACAAGCGCACTGAAG-3’; SmTSP-2 R: 5’-CCCCGTCTTTAGAGCATGAA-3’; Chimera F: 5’-CCAAAGGACTATGGCGAAAA-3’; Chimera R: 5’-GATGCTGACGCTAGTCACGA-3’. PCR measurements were conducted in triplicate. The Genbank accession numbers for Sm29 is AAC98911.1 and for TSP-2 is AAN17276.1.

### DNA vaccine protein expression in BHK-21 cells

To evaluate DNA vaccine protein expression, BHK-21 cells were grown in RPMI medium in 6-well plates and transfected using 1.0 μg of pUMVC3/Sm29, pUMVC3/SmTSP-2, pUMVC3/Chimera or the control (pUMVC3) as described above. After transfection, the cells were maintained in the RPMI medium for 16 hours at 37°C in 5% CO_2_. Then, total protein was extracted using lysis cellular buffer (50 mM Tris-HCl pH 7.4, 150 mM NaCl, 50 mM NaF, 10 mM β-Glycerophosphate, 0.1 mM EDTA, 10% glycerol, 1% Triton X-100, 1 mM sodium orthovanadate and 1:100 of protease inhibitor cocktail (Sigma, P8340). Thereafter, the samples were centrifuged at 3000 rpm for 30 seconds, and the pellet was recovered and subjected to 15% SDS-PAGE. The gels were electroblotted onto a nitrocellulose membrane according to reference [30], and the nitrocellulose membranes were blocked with phosphate buffered saline containing 5% dry milk overnight at 4°C. Subsequently, the membranes were washed three times with PBS-T (phosphate buffered saline, pH 7.2 with 0.05% Tween-20) and incubated for 1 hour at room temperature with sera of Sm29 or TSP-2 (1:200) vaccinated mice. Furthermore, the protocol was performed as described previously [19].

### Mice and parasites

C57BL/6 female mice, 6–8 weeks old, were obtained from the Federal University of Minas Gerais (UFMG) animal facility. For the sacrifice, the mice were anesthetized by an intraperitoneal injection of xylazine (12mg/Kg) and ketamine (80mg/Kg), followed by cervical dislocation. Cercariae of *S*. *mansoni* (LE strain) were maintained routinely in *Biomphalaria glabrata* snails at Rene Rachou Research Center (Fiocruz, Brazil) and prepared by exposing infected snails to light for 2 hours to induce shedding of parasites.

### Ethics statement

The protocols involving the animals used in this study were approved by the Federal University of Minas Gerais Ethics Committee on animal experimentation (CETEA No. 254/2010).

### Mice immunization

Five groups of female C57BL/6 mice (10 animals per group) were included in this study. Each group received four doses of 50 μg of purified DNA in each quadriceps muscle (100 μg total per mouse) at an interval of 15 days between each dose. One group received 100 μg of pUMVC3 alone, another received 100 μg of pUMVC3/Sm29, the third group received 100 μg of pUMVC3/SmTSP-2, the fourth group received 100 μg of pUMVC3/Chimera and the fifth group received pUMVC3/Sm29 plus pUMVC3/SmTSP-2 (50 μg of each plasmid).

### Challenge infection and worm burden recovery

Fifteen days after the final immunization, the mice were challenged with 100 cercariae (LE strain) by percutaneous exposure of the abdominal skin for 1 hr. Forty-five days after challenge, adult worms were perfused from the portal veins, as described previously [19,31]. Two independent experiments were performed to determine protection levels. The degree of protection was calculated by comparing the number of worms recovered from each vaccinated group to the respective control group as previously described [19].

### Measurement of specific anti-Sm29, anti-TSP-2 and anti-chimera antibodies

Following immunization, sera from all ten mice from each vaccinated or control group were collected at two-week intervals. Maxisorp 96-well microtiter plates (Nunc, Denmark) were coated with 25 μg/ml of the recombinant protein Sm29, TSP-2 or chimera (mix of chimera A and chimera B) in a carbonate-bicarbonate buffer, pH 9.6 for 16 hours at 4°C, then blocked for 2 hours at room temperature with 200 μl/well PBS-T (phosphate buffer saline, pH 7.2 with 0.05% Tween-20) plus 10% FBS (fetal bovine sera). The ELISA assay protocol was performed as described previously [19].

### Cytokine analysis

Cytokine experiments were performed using splenocyte cultures from individual mice immunized with pUMVC3/Sm29, pUMVC3/SmTSP-2, pUMVC3/Chimera, pUMVC3/Sm29 plus pUMVC3/SmTSP-2 or pUMVC3 as a control (n = 5 for each group). Splenocytes were isolated from individual mouse ten days after the fourth immunization and 45 days after challenge with *S*. *mansoni* and were washed twice with sterile PBS. After washing, the cells were adjusted to 1x10^6^ cells per well for IL-4, IL-5, IFN-γ and TNF-α assays in RPMI 1640 medium (Gibco) supplemented with 10% FBS, 100 U/ml of penicillin G sodium, 100 μg/ml of streptomycin sulfate, and 250 ng/ml of amphotericin B. Splenocytes were maintained in cultures of either medium alone or stimulated as follows: with recombinant Sm29 (5 μg/ml), TSP-2 (5 μg/ml), Chimera (Mix of Chimera A and B) (5 μg/ml), concanavalin A (ConA) (5 μg/ml), or LPS (1 μg/ml) as previously described [19,31,32]. For cytokine assays, polymyxin B (30 μg/mL) was added to the cultures, and this treatment completely abrogated the cytokine response to LPS as previously described [33]. Culture supernatants were collected after 24 hours for IL-4 and IL-5, after 48 hours for TNF-α and after 72 hrs for IFN-γ. Assays for the measurements of all cytokines were performed using the Duoset ELISA kit (R&D Diagnostic, Minneapolis, MN) according to the manufacturer’s directions.

### Histopathologic analysis of the liver

Following perfusion for the recovery of the schistosomes, liver sections from mice (8/group) in the control and experimental groups were collected to evaluate the effect of DNA immunization on granuloma formation. Liver sections removed from the central part of the left lateral lobe were fixed with 10% buffered formaldehyde in PBS. Histological sections were performed using a microtome at 5 μm and stained on a slide with hematoxylin-eosin. The area from each liver section (μm^2^) was calculated using KS300 software connected to a Carl Zeiss image analyzer. To perform measurements of the total area of granulomas, 20 granulomas with a single, well-defined egg from each animal were randomly chosen using a CX31 OLYMPUS light microscope with a 10× objective lens. Granuloma images were obtained using an OLYMPUS SC30 micro camera. Using ImageJ software, the areas were measured and expressed in square micrometers (μm^2^).

### Statistical analysis

Statistical analysis was performed with Student’s *t*-test for comparisons between two experimental groups using the GraphPad Prism software package (La Jolla, CA). Bonferroni adjustments were included for multiple comparisons to reduce the risk of reaching false conclusions based on chance. *P* values obtained by these methods were considered significant if they were <0.05.

## Results

### DNA vaccine gene expression in BHK-21 cells

**Fig 1:**
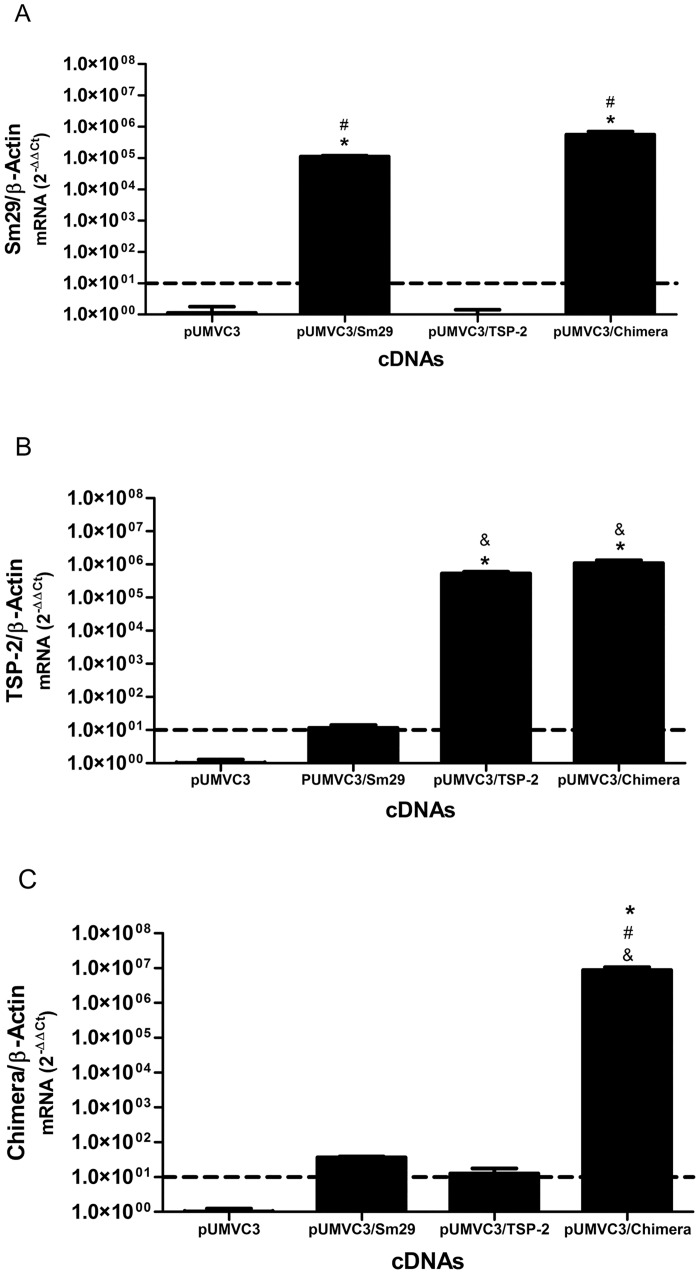
Eukaryotic mRNA expression of Sm29, TSP-2 and chimera in BHK-21 cells. RT-PCR of cells transfected with pUMVC3, pUMVC3/Sm29, pUMVC3/TSP-2 and pUMVC3/Chimera BHK-21 using specific primers for Sm29 (A), TSP-2 (B) and a chimeric region of a fusion of both (C). (*) p<0.05 compared to control group pUMVC3; (#) p<0.05 compared to group pUMVC3/TSP-2; (&) p<0.05 compared to group pUMVC3/Sm29.

**Fig 2:**
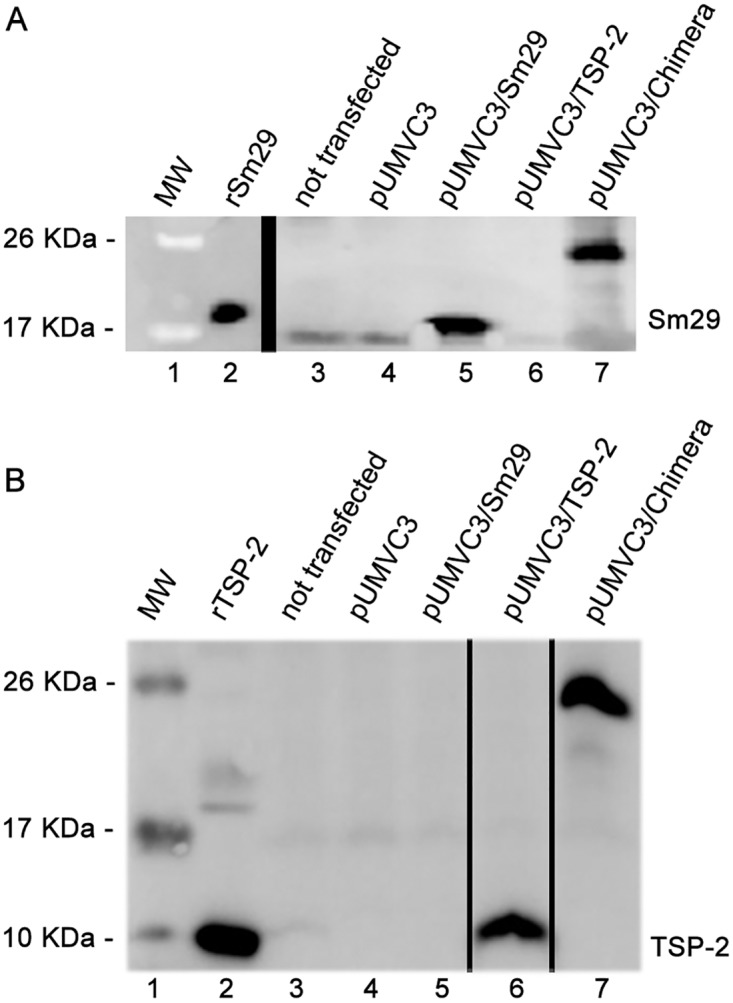
Eukaryotic protein expression of Sm29, TSP-2 and chimera in transfected BHK-21 cells. Immunoblotting using antibodies against Sm29 (A) or TSP-2 (B). Lane 1, Molecular weight (PageRuller Prestained Protein Ladder; Thermo Scientific); Lane 2, rSm29 (panel 2A) or rTSP-2 (panel 2B); Lane 3, non-transfected BHK-21 cells; Lane 4, BHK-21 cells transfected with the control group pUMVC3; Lane 5, transfected with pUMVC3/Sm29; Lane 6, transfected with pUMVC3/TSP-2; Lane 7, transfected with pUMVC3/Chimera.

The DNA vaccine constructs used in immunization experiments were analyzed for mRNA and protein expression levels in BHK-21 cells. mRNA expression was determined by real time RT-PCR analysis using specific primers for Sm29, SmTSP-2 and for a specific region of the chimera. Sm29 transcripts were detected only in extracts of cells transfected with pUMVC3/Sm29 or pUMVC3/Chimera (Fig 1A). Significant levels of SmTSP-2 mRNA were observed in extracts of pUMVC3/SmTSP2 and pUMVC3/chimera transfected cells compared to the controls (Fig 1B). When primers for the chimera were used, we detected mRNA transcripts only in extracts from the pUMVC3/chimera DNA vaccine (Fig 1C). These results indicate that DNA vaccines were expressed at the mRNA level. For protein expression, BHK-21 extracts were evaluated by immunoblotting analysis using antibodies to Sm29 or SmTSP-2 proteins. When antibodies against Sm29 were used, we detected a specific band of 18 kDa only in the extracts of cells transfected with pUMVC3/Sm29 (Fig 2A). Additionally, we observed a band of 12 kDa in the extracts of cells transfected with pUMVC3/TSP-2 when serum containing antibodies against TSP-2 (Fig 2B) was used. Furthermore, a band of approximately 25 kDa was detected only in extracts of cells transfected with pUMVC3/Chimera when either Sm29 or the TSP-2 sera (Fig 2A and 2B) were used. These results indicate that DNA vaccines were expressed at the protein level.

### Determination of DNA vaccine efficacy

In order to assess the ability of DNA vaccination with pUMVC3/Sm29, pUMVC3/SmTSP-2, pUMVC3/Chimera or pUMVC3/Sm29 plus pUMVC3/TSP-2 to induce protection in the murine model, the mice were challenged with 100 cercariae of *S*. *mansoni* 15 days after the last immunization, and the worm burden recovery was determined. Immunization with the plasmids pUMVC3/Sm29, pUMVC3/TSP-2, pUMVC3/Chimera or pUMVC3/Sm29 plus pUMVC3/TSP-2 induced 17–22%, 22%, 31–32% or 24–32% worm reduction, respectively, when compared to the control group (Table 1). Additionally, in the first trial, the mice immunized with pUMVC3/Chimera showed an enhanced protection against challenge infection when compared to the group immunized with pUMVC3/Sm29 alone; in the second trial, even with a higher protection elicited by pUMVC3/Chimera, this difference was not significant. This result validates the use of chimeras as a strategy to potentiate vaccine efficacy against *S*. *mansoni*.

**Table 1:** Protection level induced by DNA immunization.

First trial ^a^	Worms recovered ± SD	% of protection
pUMVC3	79.3 ± 4.8	-
pUMVC3/Sm29	65.8 ± 4.5	17 *
pUMVC3/TSP-2	61.9 ± 2.8	22 *
pUMVC3/Chimera	54.7 ± 5.8	31 * ^#^
pUMVC3/Sm29 + pUMVC3/TSP-2	60.3 ± 5.8	24 *
Second trial ^a^		
pUMVC3	45.0 ± 7.8	-
pUMVC3/Sm29	34.9 ± 5.2	22 *
pUMVC3/TSP-2	35.1 ± 7.0	22 *
pUMVC3/Chimera	30.7 ± 6.9	32 *
pUMVC3/Sm29 + pUMVC3/TSP-2	30.5 ± 5.9	32*

* p<0.05 compared to pUMVC3 control

^#^ p<0.05 compared to pUMVC3/Sm29

^a^ Ten animals per group in each independent experiment

### Antibody response to DNA vaccination

**Fig 3:**
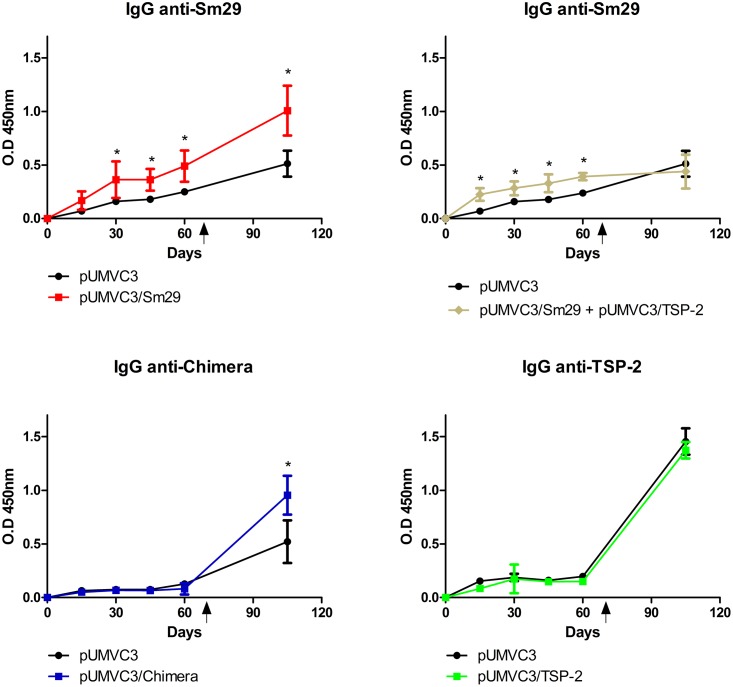
Total IgG antibody levels anti-Sm29, anti-Chimera and anti-Sm-TSP2 after DNA vaccination. Levels of anti-Sm29, anti-Chimera and anti-Sm-TSP2 antibodies in the sera of mice immunized with the DNA vaccines and challenged were measured. The arrows represent the time of challenge infection at day 67^th^. Significant differences from the sera of mice immunized with the appropriate DNA vaccines compared to the pUMVC3 control group are denoted by an asterisk (*) for p<0.05.

To evaluate the level of specific IgG antibodies against Sm29, TSP-2 and chimera sera from the vaccinated mice were tested by ELISA (Fig 3). Prior to challenge, when the sera were assessed, only the sera of mice immunized with pUMVC3/Sm29 and plasmids pUMVC3/Sm29 and pUMVC3/TSP-2 together showed higher titers of anti-Sm29 when compared with the control group pUMVC3. After immunization, when the levels of antibodies against the recombinant TSP-2 were evaluated, no differences were observed in sera of vaccinated mice compared to the plasmid alone control group (S1 Fig). After challenge, the levels of total IgG anti-Sm29 and anti-chimera in pUMVC3/Sm29 and pUMVC3/chimera vaccinated groups, respectively, were higher when compared to the plasmid alone control group (Fig 3).

### Cytokine profile produced by spleen cells of mice immunized with DNA vaccines

**Fig 4:**
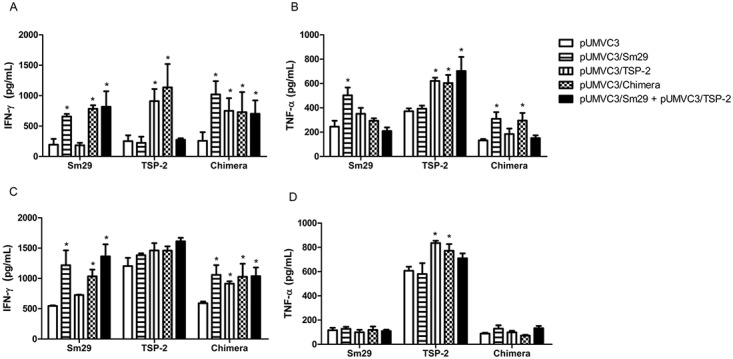
Cytokine profile of mice immunized with the DNA vaccines. Levels of IFN-γ (A) and TNF-α (B) were measured in the supernatants of spleen cells of mice immunized with the DNA vaccines. Levels of IFN-γ (C) and TNF-α (D) were detected in splenocytes of mice vaccinated and challenged and the cells were restimulated with recombinant Sm29, TSP-2 or chimera. Significant differences from stimulated spleen cells of mice immunized with the pUMVC3 control are denoted by an asterisk (*) for p<0.05.

Cytokine production by spleen cells of immunized mice was stimulated with recombinant Sm29, Sm-TSP-2 or chimera proteins and measured both after the fourth immunization and after the challenge infection. After the fourth dose of vaccines, high levels of IFN-γ were detected in cell supernatants of mice immunized with pUMVC3/Sm29, pUMVC3/Chimera and pUMVC3/Sm29 + pUMVC3/TSP-2 when stimulated with the Sm29 recombinant, in cell supernatants of mice immunized with pUMVC/TSP-2 and pUMVC3/Chimera when stimulated with the TSP-2 recombinant, as well as of all immunized mice when stimulated with the recombinant chimera, when compared to the control group pUMVC3 (Fig 4A). When the levels of IFN-γ production in the supernatants of cells of mice immunized and infected with cercariae of *S*. *mansoni* were evaluated, higher production was observed only in the cells of mice immunized with pUMVC3/Sm29, pUMVC3/Chimera and pUMVC3/Sm29 + pUMVC3/TSP-2 when stimulated with the Sm29 recombinant, as well as in the supernatants of all immunized mice when stimulated with the recombinant chimera (Fig 4C). When the levels of TNF-α were evaluated after the fourth dose of vaccines, we observed significant production of this cytokine in the supernatant of cells of mice immunized with pUMVC3/Sm29 when stimulated with Sm29 recombinant, in the supernatants of cells of mice immunized with pUMVC3/TSP-2, pUMVC3/Chimera and pUMVC3/Sm29 + pUMVC3/TSP-2 when stimulated with the TSP-2 recombinant, and in the supernatants of mice immunized with pUMVC3/Sm29 and pUMVC3/Chimera when stimulated with recombinant chimera compared with the pUMVC3 control group (Fig 4B); When the levels of TNF-α in cultures of immunized and challenged mice were evaluated, only the supernatants of mice immunized with pUMVC3/TSP-2 and pUMVC3/Chimera when stimulated with the recombinant TSP-2 showed higher levels when compared to the control group (Fig 4D). When the levels of IL-4 and IL-5 were evaluated, no differences were observed in any stimuli of any group tested in relation to the control group (data not shown).

### Histological analysis

**Fig 5:**
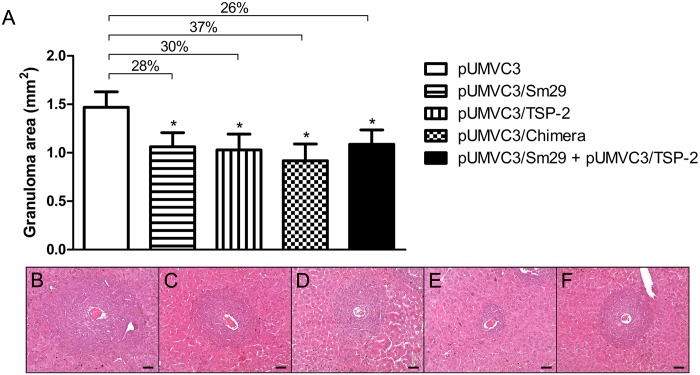
Granuloma area reduction and representative granulomas of mice immunized with the DNA vaccines and challenged with *S*. *mansoni* cercariae. (A) Graph showing the granuloma area in liver of vaccinated mice. Representative granulomas of (B) pUMVC3; (C) pUMVC3/Sm29; (D) pUMVC3/TSP-2; (E) pUMVC3/Chimera; (F) pUMVC3/Sm29 + pUMVC3/TSP-2 are demonstrated. The scale bar corresponds to 50 μm. Significant differences from mice immunized with the pUMVC3 control are denoted by an asterisk (*) for p<0.05, and the percent reductions are shown.

The livers of mice immunized and challenged with 100 cercariae of *S*. *mansoni* were collected, and the areas of granulomas were evaluated. All mice immunized with the DNA vaccines showed a significant reduction in granuloma area when compared with the control group (Fig 5B–5F). The mice immunized with pUMVC3/Sm29, pUMVC3/TSP-2, pUMVC3/Chimera and the pUMVC3/Sm29 + pUMVC3/TSP-2 mixture exhibited 28%, 30%, 37% and 26% granuloma area reduction when compared to the control group, respectively (Fig 5A).

## Discussion

The main protective immunological response in murine schistosomiasis is believed to develop against migrating schistosomula in the lungs [9,34], and the strategic localization of the proteins Sm29 and SmTSP-2 in the tegument of adult worms and schistosomula of *S*. *mansoni* together with high levels of protection when administered in a recombinant form render them powerful candidates for the development of an efficient vaccine against *S*. *mansoni* [18,19]. Therefore, the main goal of this study was to evaluate the potential of DNA vaccines encoding the Sm29 or SmTSP-2 genes, a chimera of both and joint administration of the two.

To evaluate whether the DNA vaccines could be transcribed and translated in eukaryotic cells, BHK-21 cells were transfected with plasmids encoding the genes of interest, and we detected the levels of mRNA and protein by real time RT-PCR and western blot, respectively. These results indicate that Sm29, TSP-2 and chimeric DNA vaccines were transcribed, and the masses of the proteins produced were of the expected sizes of 18, 12 and 25 kDa, respectively. The confirmation of protein expression of the DNA vaccines avoids the possibility of false negatives in vaccination experiments.

The DNA vaccines pUMVC3/Sm29, pUMVC3/TSP-2, pUMVC3/Chimera and joint administration of the pUMVC3/Sm29 and pUMVC3/SmTSP-2 plasmids resulted in worm burden reduction when compared to control group pUMVC3 of 17–22%, 22%, 31–32% and 24–32%, respectively. The DNA vaccines pUMVC3/Sm29 and pUMVC3/TSP-2 showed reduced levels of protection when compared with the recombinant protein vaccines for Sm29 or TSP-2 formulated in Freund’s adjuvant, which achieved 51% and 57% reductions, respectively [18,19], but are very similar when compared with the Sm29 or TSP-2 recombinant when administered in CpG alum adjuvant (20% and 25–27% of protection, respectively) [23,28]. Probably, the higher levels of recombinant protein vaccine protection when compared to DNA vaccine protection are due to the use of a Freund’s adjuvant in the formulation of protein recombinant vaccines because the DNA vaccine formulations have unmethylated CpG motifs in their plasmid as the only adjuvant. However, the use of Freund’s adjuvant is limited because it is too toxic for human use [35]. The use of chimeras is a strategy to optimize the immunological response against schistosomiasis [22,23,28]. The use of a plasmid that encodes a chimera sequence of Sm29 and TSP-2 is responsible for an improvement in the levels of protection against *S*. *mansoni* when compared to the group immunized with pUMVC3/Sm29. This enhanced protection may be due to different conformations achieved by the chimera produced by the host cells, but future studies are required to better clarify this phenomenon. This indicates the potential of this strategy for enhancing the protective response against schistosomiasis. When the plasmids encoding the Sm29 and TSP-2 sequences were used simultaneously, no significant increase in the protective response was observed in relation to the group pUMVC3/Sm29 or pUMVC3/TSP-2 in any trial. In the immunization schedule of the group pUMVC3/Sm29 + pUMVC3/TSP-2, 50 μg of each individual plasmid was administered, whereas, in all other single plasmid immunizations the amount of injected plasmid was always 100 μg. Therefore, as DNA vaccines requires relative greater amounts of plasmids to be effective in the intramuscular route of administration [14,36], we believe that less quantity of each individual plasmid administered in the pUMVC3/Sm29 + pUMVC3/TSP-2 group was not sufficient to improve the efficacy of each single plasmid immunization, as observed for pUMVC3/Chimera.

When the levels of antibodies elicited by the DNA immunization were evaluated, only in the sera of mice immunized with the pUMVC3/Sm29 or pUMVC3/Sm29 together with pUMVC3/TSP-2 that we observed higher levels of antibodies against Sm29 when compared to pUMVC3 control group. Unexpectedly, after immunization we did not detect IgG anti-rSm29 in pUMVC3/Chimera immunized mice. However, after challenge the levels of total IgG anti-chimera in pUMVC3/Chimera vaccinated mice were higher than the levels observed in the pUMVC3 control group. The protein produced by the immunization with pUMVC3/Chimera is composed by the sequence of Sm29 and the entire extracellular loop-2 (ECL-2) of Sm-TSP-2. Probably, the protein produce by the DNA immunization undergoes post-translational modifications and has a more complex and different conformation compared to the recombinant Sm29 and TSP-2. Sm29 has three possible sites of O-glycosilation (Thre39, Thre132 and Thre 133) and two possible sites of N-glycosilation (Asn58 and Asn115) and Sm-TSP-2 undergo post-translational modification in which palmitate is bound to the membrane proximal cysteine residues and associates with cholesterol-rich domains [24, 25]. Therefore, antibodies against glycoproteins containing sugar residues produced by DNA vaccination may not recognize rSm29 or rSm-TSP-2 produced in bacteria. Additionally, due to this more complex chimera produced some conformational B-cell epitopes may be changed in this protein and the antibodies produced no longer recognize rSm29 or rTSP-2. However, even without an apparent humoral response against rSm29 or rSm-TSP-2, the animals immunized with pUMVC3/Chimera possess the ability to mount a strong cellular immunological response, as seen by the high production of the cytokines IFN-γ and TNF-α when stimulated with the recombinant Sm29, TSP-2 or the recombinant chimera. Furthermore, immunized mice were not able to mount a significant humoral response against the recombinant TSP-2 compared to plasmid alone control group. As the ECL-2 from tetraspanin-2, is a protein that naturally exists in *Schistosoma mansoni* as a strong coordinated molecule mainly orientated by disulfide bridges along the structure [18,24], it is possible that antibodies elicited against Sm-TSP-2 produced *in vivo* by DNA vaccination together with post-translational modifications that happens during mammalian protein expression as mentioned above may result in antibodies that do not recognize the recombinant form of TSP-2 produced in bacteria as observed in the ELISA assay. However, when we performed western blot analysis by chemiluminescence using concentrated antibodies from pUMVC3/TSP-2 immunized and challenged mice in protein extracts from BHK-21 transfected cells, we showed a significant binding of circulating antibodies to TSP-2 (S2 Fig).

The vaccines tested in this study induced a Th1 profile, which was shown by the production of IFN-γ and TNF-α and no significant IL-4 or IL-5. Several studies using recombinant protein or DNA vaccine platforms have shown results relating the Th1 profile of cytokines induced by the immunizations to protective immunity against *S*. *mansoni*, such as Sm-p80, Sm29, stomatin-like protein-2 and rp22, which achieved higher levels of protection. Additionally, these results were associated with increases in the production of IFN-γ and TNF-α [15,19,37–39]. The involvement of IFN-γ in protective immunity against *S*. *mansoni* is well described. Several studies have shown that mice that are deficient in the gene encoding the sequence for IFN-γ or the use of antibodies against IFN-γ showed a reduced immune response when immunized with irradiated cercariae [9,34]. The protective response mediated by IFN-γ probably occurs against migrating schistosomula present in the lungs. This mainly involves the formation of macrophage and lymphocyte foci around the worms, hindering their movement in the lungs and moving them to the airways with consequent elimination of these parasites [9,34]. Another potential mechanism mediated by IFN-γ is the activation of macrophages to kill worms in a nitric oxide-dependent pathway [9,40].

Previous studies have demonstrated the potential for recombinant Sm29 and TSP-2 to reduce liver pathology [18,19,28]. Our results are in agreement with these studies because our DNA vaccines decreased the granuloma area in all groups evaluated. The pathology characterized by granuloma formation around the eggs in the murine model of schistosomiasis is related to Th2 cytokines, such as IL-4 and IL-13 [40–42]. IL-13 and IL-4 induce the synthesis of collagen and fibrosis in a mechanism mediated by the enzyme arginase, which is responsible for the conversion of L-arginine to L-ornithine, a molecule essential to collagen formation. Components of the Th1 response, such as IFN-γ and TNF-α, induce the expression of inducible nitric oxide synthase (iNOS), an enzyme that converts L-arginine to L-hydroxy-arginine, which inhibits the synthesis of collagen, and hence, decreases granuloma area [40–42]. Therefore, we believe that the levels of granuloma reduction achieved with the DNA vaccination may be related to the potential for our vaccines to induce high levels of IFN-γ and TNF-α.

## Conclusion

In summary, we demonstrate that DNA vaccines encoding the sequences of Sm29 and Sm-TSP-2, or a chimeric form of both, elicit an immunological response characterized by the production of Th1 cytokines, moderate levels of antibodies and reduced granulomas. However, the DNA vaccines studied here confer only partial protection in a murine model of schistosomiasis. These findings support the idea of using the candidates Sm29 and Sm-TSP-2, alone or together, in a multiple DNA vaccine against schistosomiasis, but, further studies to improve the efficacy are needed.

## Supporting Information

S1 Fig Anti-TSP-2 antibodies. Levels of anti-TSP-2 antibodies in the sera of mice immunized with the DNA vaccines and compared with the pUMVC3 control group.
(TIF)

S2 Fig Antibody binding to schistosome proteins produced by transfected BHK-21 cells with the DNA vaccines. BHK-21 cells were transfected with the plasmids pUMVC3, pUMVC3/Sm29 and pUMVC3/TSP-2 and incubated with sera of mice immunized with pUMVC3/Sm29 (B) or pUMVC3/TSP-2 (C) and 45 days after challenged. The sera of mice immunized with the recombinant Sm29 and TSP-2 were used as positive controls (A). MW: Molecular weight (PageRuller Prestained Protein Ladder; Thermo Scientific).
(TIF)
