# Supplementary figures and images for: DNA Vaccine Encoding the Chimeric Form of Schistosoma mansoni Sm-TSP2 and Sm29 Confers Partial Protection against Challenge Infection
Source: PLoS One. 2015 May 5;10(5):e0125075. doi: 10.1371/journal.pone.0125075 (PMC4420270; doi:10.1371/journal.pone.0125075)

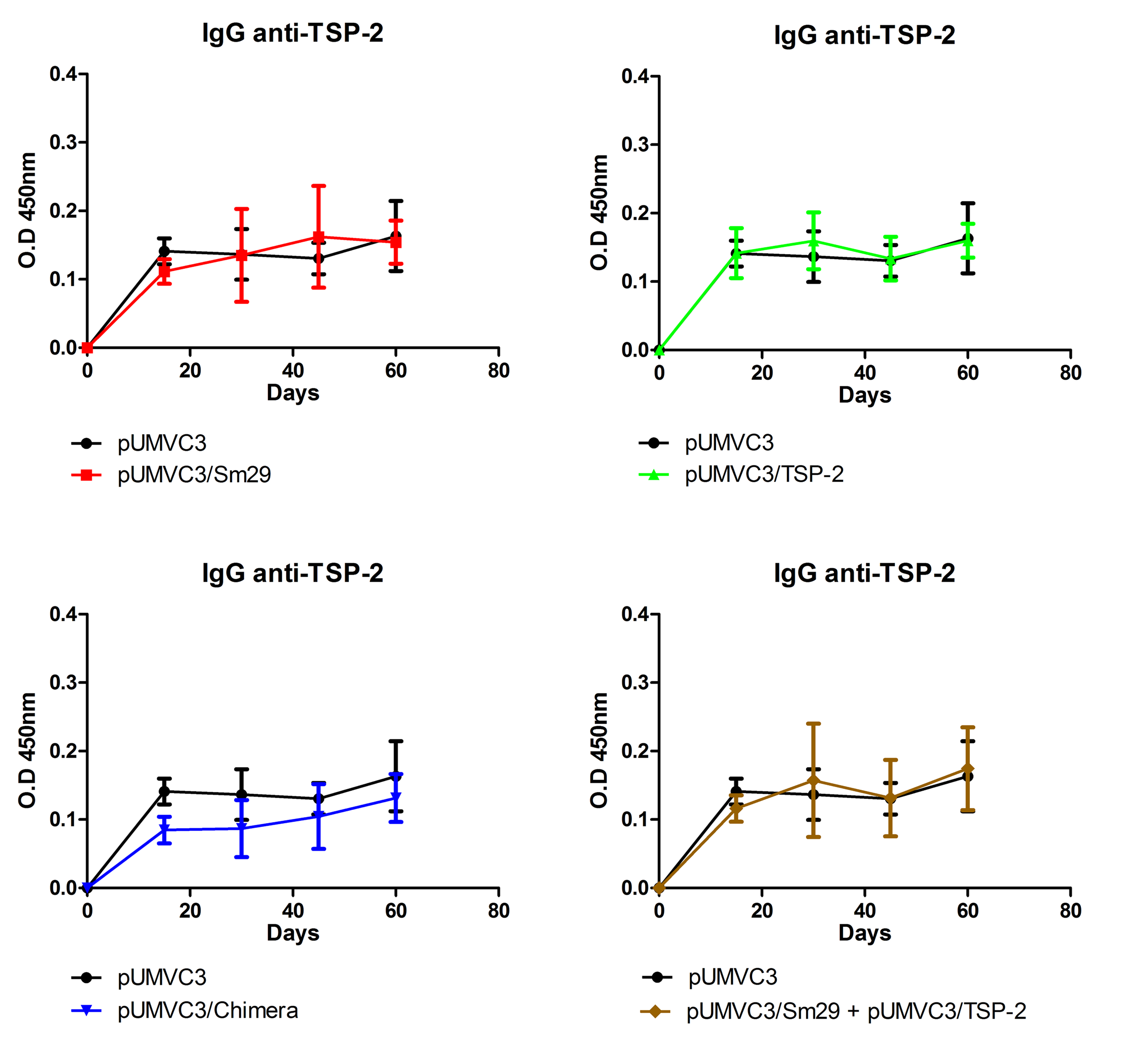

Supplement: S1 Fig — Levels of anti-TSP-2 antibodies in the sera of mice immunized with the DNA vaccines and compared with the pUMVC3 control group. (TIF) [file pone.0125075.s001.tif]

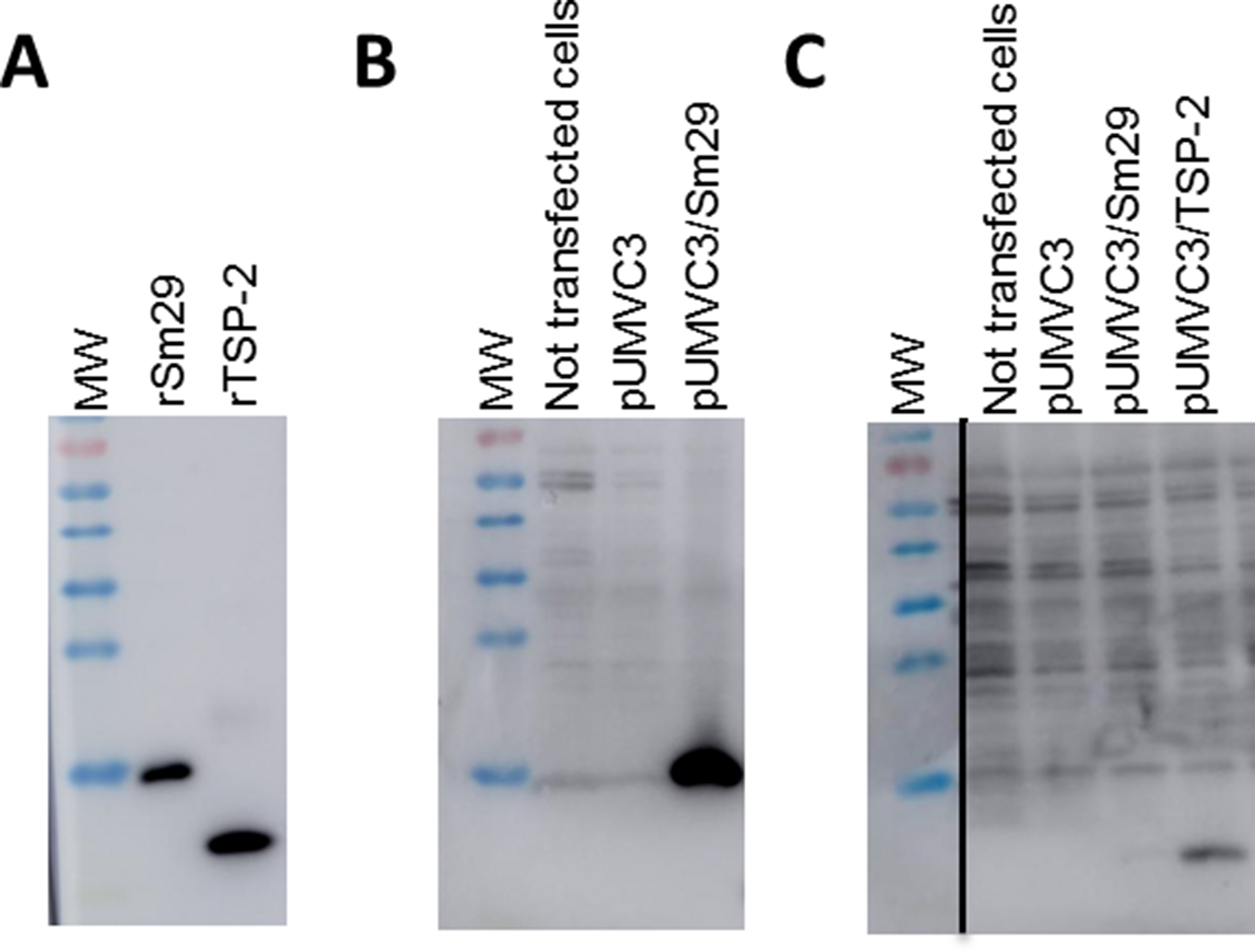

Supplement: S2 Fig — BHK-21 cells were transfected with the plasmids pUMVC3, pUMVC3/Sm29 and pUMVC3/TSP-2 and incubated with sera of mice immunized with pUMVC3/Sm29 (B) or pUMVC3/TSP-2 (C) and 45 days after challenged. The sera of mice immunized with the recombinant Sm29 and TSP-2 were used as positive controls (A). MW: Molecular weight (PageRuller Prestained Protein Ladder; Thermo Scientific). (TIF) [file pone.0125075.s002.tif]
